# Left Atrial Mechanical Function and Global Strain in Hypertrophic Cardiomyopathy

**DOI:** 10.1371/journal.pone.0157433

**Published:** 2016-06-23

**Authors:** Kyung-Jin Kim, Hong-Mi Choi, Yeonyee E. Yoon, Hack-Lyoung Kim, Seung-Pyo Lee, Hyung-Kwan Kim, Yong-Jin Kim, Goo-Yeong Cho, Joo-Hee Zo, Dae-Won Sohn

**Affiliations:** 1 Department of Internal Medicine, Seoul National University College of Medicine, Seoul, Republic of Korea; 2 Department of Internal Medicine, Cardiovascular Center, Seoul National University Hospital, Seoul, Republic of Korea; 3 Division of Cardiology, Department of Internal Medicine, Seoul National University Bundang Hospital, Seongnam-si, Gyeonggi-do, Republic of Korea; 4 Division of Cardiology, Department of Internal Medicine, Seoul National University Boramae Medical Center, Seoul, Republic of Korea; Loyola University Chicago, UNITED STATES

## Abstract

**Background:**

Atrial fibrillation is the most common arrhythmia and is associated with adverse outcomes in hypertrophic cardiomyopathy (HCM). Although left atrial (LA) remodeling and dysfunction are known to associate with the development of atrial fibrillation in HCM, the changes of the LA in HCM patients remain unclear. This study aimed to evaluate the changes in LA size and mechanical function in HCM patients compared to control subjects and to determine the characteristics of HCM associated with LA remodeling and dysfunction.

**Methods:**

Seventy-nine HCM patients (mean age, 54 ± 11 years; 76% were men) were compared to 79 age- and sex-matched controls (mean age, 54 ± 11 years; 76% were men) and 20 young healthy controls (mean age, 33 ± 5 years; 45% were men). The LA diameter, volume, and mechanical function, including global strain (ε), were evaluated by 2D-speckle tracking echocardiography. The phenotype of HCM, maximal left ventricular (LV) wall thickness, LV mass, and presence and extent of late gadolinium enhancement (LGE) were evaluated with cardiac magnetic resonance imaging.

**Results:**

HCM patients showed increased LA volume index, impaired reservoir function, and decreased LA ε compared to the control subjects. When we divided the HCM group according to a maximal LA volume index (LAVI_max_) of 38.7 ml/m^2^ or LA ε of 21%, no significant differences in the HCM phenotype and maximal LV wall thickness were observed for patients with LAVI_max_ >38.7 ml/m^2^ or LA ε **≤**21%. Conversely, the LV mass index was significantly higher both in patients with maximal LA volume index >38.7 ml/m^2^ and with LA ε **≤**21% and was independently associated with LAVI_max_ and LA ε. Although the LGE extent was increased in patients with LA ε **≤**21%, it was not independently associated with either LAVI_max_ or LA ε.

**Conclusions:**

HCM patients showed progressed LA remodeling and dysfunction; the determinant of LA remodeling and dysfunction was LV mass index rather than LV myocardial fibrosis by LGE-magnetic resonance imaging.

## Introduction

Atrial fibrillation is the most common arrhythmia in patients with hypertrophic cardiomyopathy (HCM), occurring in about one fifth of all HCM patients, which is four times the frequency expected in the general population [1–3], and causing substantial morbidity and mortality by promoting progressive heart failure and increasing the risk for embolic stroke [2–4]. Therefore, early recognition of susceptibility to atrial fibrillation would be advantageous for longitudinal surveillance and timely prophylactic intervention and management strategies in HCM patients. In such patients, the left atrium (LA) has been shown to have an increased size and decreased mechanical function, especially in the advanced stage [5, 6]. Moreover, atrial fibrillation is more prevalent in patients who demonstrate LA remodeling and dysfunction [2, 4, 7]. Recently, there has been increasing interest in LA strain analysis using two-dimensional (2D) speckle-tracking echocardiography to quantify the magnitude of atrial deformation [8–11]. Previous studies have reported that the LA global longitudinal strain (ε) is decreased in patients with paroxysmal atrial fibrillation compared with normal control subjects [10] and that decreased LA ε is associated with atrial fibrillation progression to a persistent or permanent stage [8] and with recurrence after catheter ablation [12]. However, little is currently known regarding the changes in LA ε in patients with HCM.

Cardiac magnetic resonance imaging (MRI) has emerged as a useful adjunctive imaging modality for the diagnosis and risk stratification of HCM [13–15]. Cardiac MRI has the unique capability of acquiring tomographic images with high spatial and temporal resolution, and with excellent tissue contrast, but without limitations associated with either the imaging window or imaging plane [13]. In addition, late gadolinium enhancement (LGE)-MRI allows noninvasive identification and quantification of myocardial fibrosis, which is associated with increased morbidity and mortality in HCM [15]. However, to date, little is known with regard to the cardiac MRI characteristics of HCM associated with LA remodeling and dysfunction. Accordingly, in the present study, we tried to determine the LA remodeling and functional changes, including LA ε, in HCM by comparing HCM patients with age- and sex-matched control subjects and with young healthy subjects. Furthermore, we also evaluated the characteristics of HCM associated with increased LA size and decreased LA ε.

## Materials and Methods

### Study population

**Fig 1:**
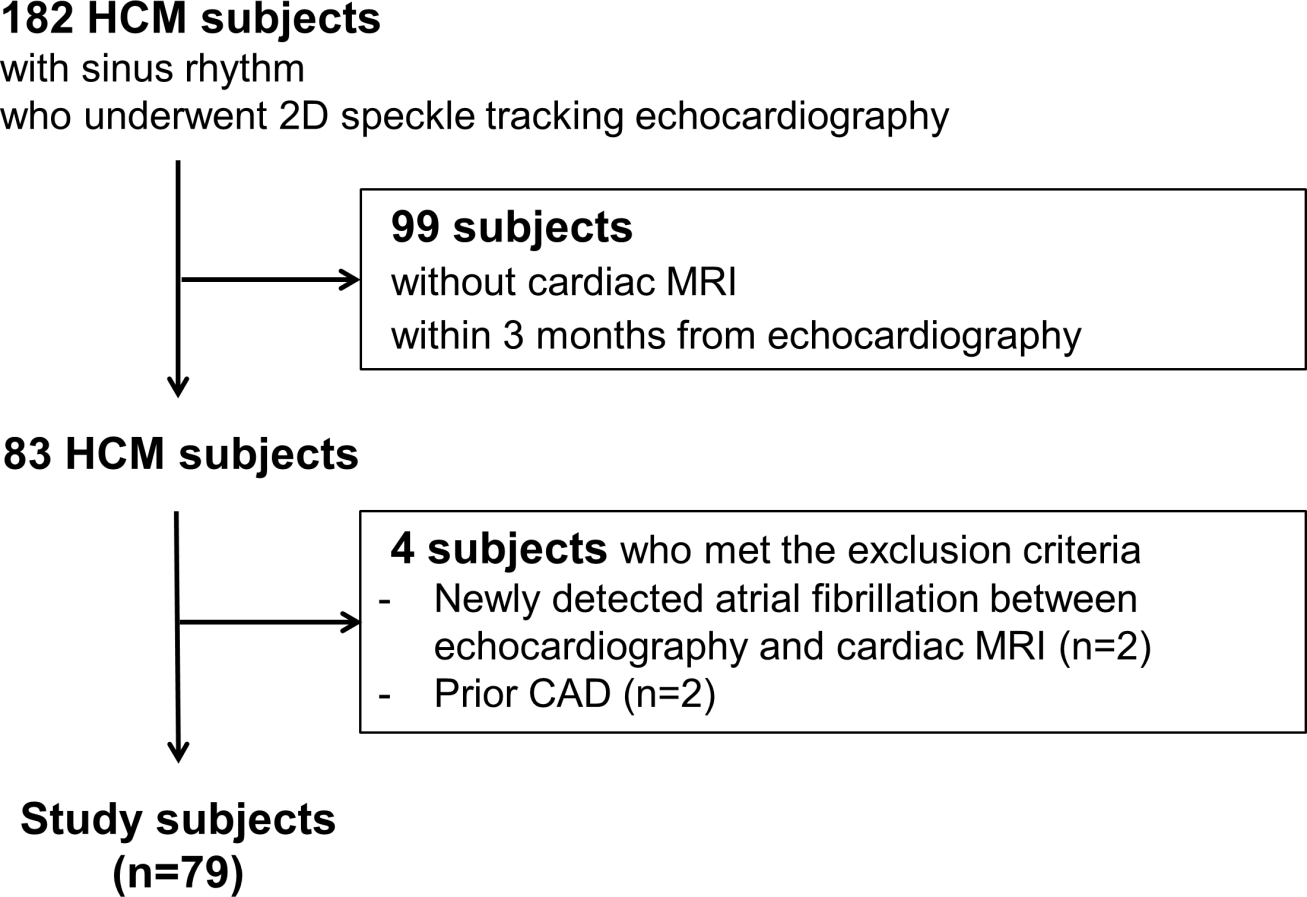
Flow chart of the study population. Abbreviations: HCM, hypertrophic cardiomyopathy; MRI, magnetic resonance imaging; CAD, coronary artery disease.

The medical records of 182 consecutive adult patients with HCM and with sinus rhythm without a history of atrial fibrillation who underwent 2D speckle tracking echocardiography at Seoul National University Bundang Hospital between 2009 and 2013 were retrospectively reviewed. HCM diagnosis was established by the presence of left ventricular (LV) hypertrophy (LV wall thickness ≥15 mm) on echocardiography, associated with a non-dilated LV chamber, in the absence of other cardiac or systemic diseases explaining the observed hypertrophy [16]. Among these, 83 patients who underwent both 2D speckle tracking echocardiography and cardiac MRI within 3 months were assessed for eligibility. Subsequently, we excluded patients with newly diagnosed atrial fibrillation between echocardiography and cardiac MRI (n = 2) and patients with a prior coronary artery disease, defined as prior myocardial infarction, prior coronary revascularization, or coronary artery disease on prior catheterization (n = 2). Thus, the remaining 79 patients (mean age, 54 ± 11 years; 76% were men) formed the study cohort (Fig 1).

For comparison of the LA size and mechanical function in HCM patients to healthy population, we also retrospectively formed two control groups: age- and sex-matched control group and young healthy control group. The age- and sex-matched control group consisted of 79 healthy subjects with similar age and sex (mean age, 54 ± 11 years; 76% were men) who were randomly selected from the subjects who volunteered for general routine health evaluation and echocardiography. The young healthy control group consisted of 20 young healthy subjects (mean age, 33 ± 5 years; 45% were men) who volunteered for both echocardiography and cardiac MRI. None of the controls had any cardiovascular disease or systemic disease or any other risk factors, and had sinus rhythm. The institutional ethics committee of Seoul National University Bundang Hospital approved this retrospective study and waived of the requirement for both written and verbal informed consent from the entire study subjects including control subjects due to the retrospective nature of the evaluation without the risk of harm to study subjects. Patient records/information was anonymized and de-identified prior to analysis.

### Transthoracic echocardiography

A Vivid 7 ultrasound system (GE Vingmed Ultrasound AS, Horten, Norway) was utilized for the transthoracic echocardiographic examination. All images and measurements were acquired from the standard views, according to the guidelines of the American Society of Echocardiography [17–19] and were digitally stored for offline analysis. As described in detail previously [10], the LA maximum anterior-posterior (A-P) diameter was measured in the parasternal long-axis view. The following LA volumes were measured using a biplane area-length method from the apical 4-chamber and 2-chamber view and were indexed according to the body surface area: maximum LA volume index (before mitral valve opening) (LAVI_max_), pre-A LA volume index (before atrial contraction) (LAVI_pre-A_), and minimum LA volume index (after atrial contraction) (LAVI_min_). The LA expansion index (%) and active emptying fraction (%) were calculated as: [(LAVI_max_−LAVI_min_) / LAVI_min_] × 100% and [(LAVI_pre-A_−LAVI_min_) / LAVI_pre-A_] × 100% [10].

Global LA myocardial longitudinal strain (ε) during ventricular systole was measured by 2D speckle tracking echocardiography, as previously described [8, 10]. Gray scale images of the apical 4-chamber view were obtained with frame rates of 50–80 Hz. All recordings were processed with speckle-tracking software (EchoPAC; GE Vingmed Ultrasound AS), allowing offline semi-automated speckle-based strain analysis. Briefly, at the time of the end-systolic phase, the lines were traced manually along the LA endocardium. An additional epicardial line, which was generated automatically by the software, created a region of interest. After manually adjusting the shape of the region of interest, the LA ε during the whole cardiac cycle was calculated [12, 20].

### Cardiac MRI

MR images were obtained by using a 1.5-T MR system (Intera CV release 10; Philips Healthcare, Amsterdam, the Netherlands) with five-channel cardiac coils. All images were acquired with electrocardiographic gating and breath-holding. Steady-state free-precession cine-MR images were obtained for each patient, including vertical long-axis images, four-chamber view images, and a set of short-axis images covering the entire LV. The sequence parameters were as follows: field of view: 350–400 mm, repetition time/echo time: 3.0–3.6/1.5–1.8 ms, flip angle: 60°, slice thickness: 8 mm. Fifteen minutes after intravenous administration of 0.2 mmol/kg of gadodiamide (Omniscan; GE Healthcare), an inversion recovery-prepared, T1-weighted, gradient-echo sequence was used to obtain LGE-MRI in the same planes as the cine images. The LGE imaging parameters were as follows: field of view: 350–400 mm, repetition time/echo time: 4.5–4.6/1.3–1.5 ms, flip angle: 15°, inversion time: 200–300 ms, slice thickness: 8 mm. The inversion time was adjusted to nullify the signal of the normal myocardium.

Imaging data were analyzed using a commercially available post-processing workstation (Mass; Medis, Leiden, the Netherlands). Endocardial and epicardial contours were prescribed manually on the short-axis cine-MRI of the LV at end-diastole and end-systole to obtain the LV volumes, mass, and ejection fraction. The presence and pattern of LGE on contrast-enhanced MRI were interpreted by the consensus of two observers blinded to the patient history and clinical outcome. The LGE mass was quantified using a threshold of 6 standard deviations above the mean signal intensity for the normal nulled myocardium [21]. Summing of the LGE mass of all slices yielded the total mass of the LGE, and the extent of LGE was expressed as a percentage of the total LV mass (the % LV mass with LGE). For statistical analysis, the LGE score and extent were divided into quartiles: LGE extent of 0%, 1–4%, 5–12%, and ≥13%; LV mass index <56 g/m^2^, 57–71 g/m^2^, 72–83 g/m^2^, and ≥84 g/m^2^.

### Statistical analysis

The clinical, echocardiographic, and MRI parameters of the HCM patients, age- and sex-matched control subjects, and young healthy control subjects are reported. Continuous variables are expressed as the means and standard deviations and categorical variables are expressed as proportions. Comparison of continuous variables was performed with the paired t-test between HCM patients and age- and sex-matched control subjects and with Student’s t-test between HCM patients and young healthy control subjects. Categorical variables were compared using the χ2 test or Fisher’s exact test, as appropriate. Between-group differences by HCM phenotype were compared by one-way analysis of variance (ANOVA) followed by Bonferroni’s post-hoc test. Further, we also evaluated the characteristics of HCM patients by two-group comparison according to the median values of LAVI_max_ and LA ε. Univariate linear regression analyses were performed to examine the effects of various characteristics of HCM on LAVI_max_ and LA ε. Covariates obtaining a *P* value <0.2 in the univariate analyses were included in the multivariate linear regression analyses. For all analyses, a two-sided *P* value of less than 0.05 was considered to represent a statistically significant difference. All analyses were performed using SPSS version 20.0 (IBM, Chicago, IL)

## Results

**Fig 2:**
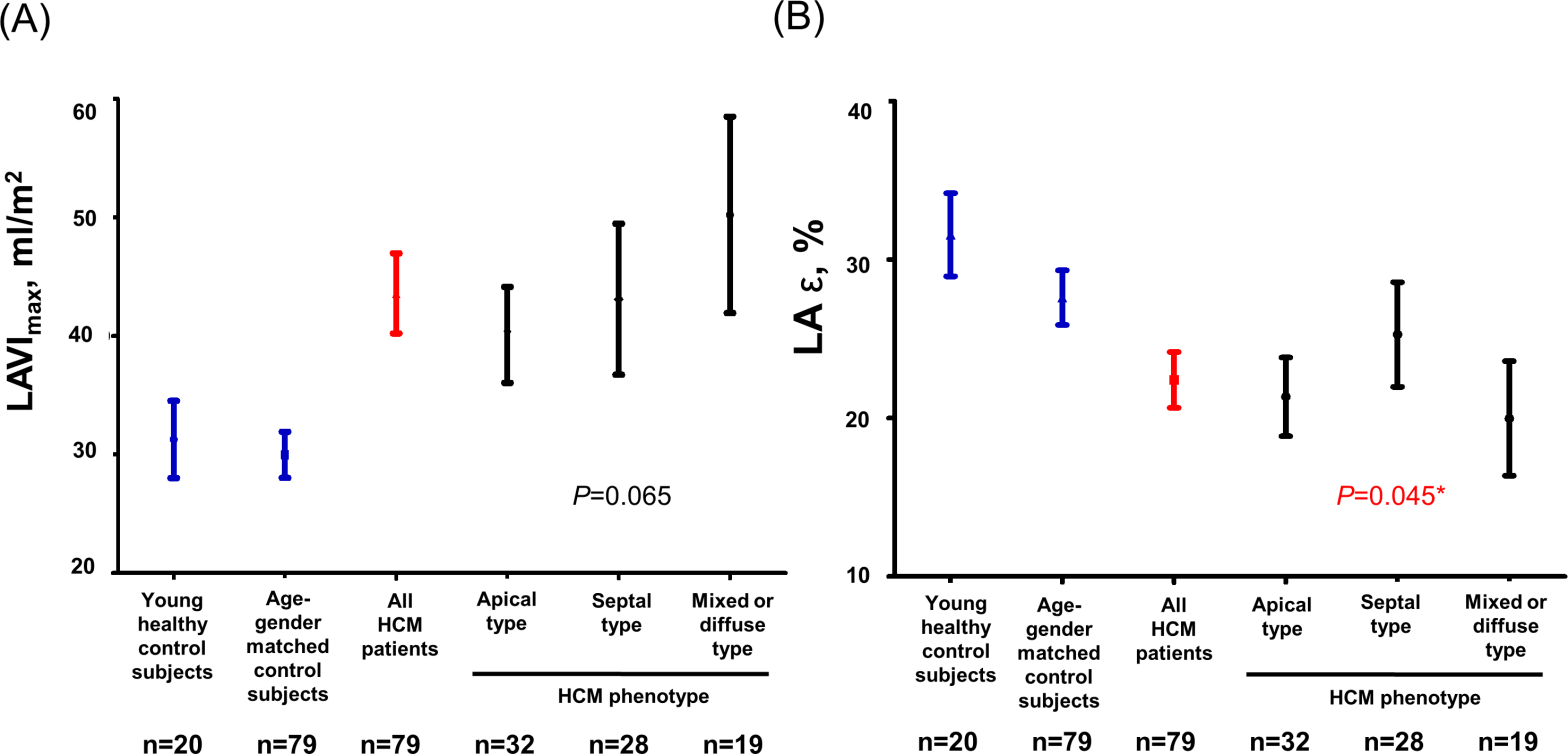
Maximal left atrial volume index (A) and global longitudinal strain (B) in young healthy control subjects, age- and sex-matched control subjects, overall hypertrophic cardiomyopathy patients, and each group of hypertrophic cardiomyopathy by the phenotype. Abbreviations: LAVI_max_, maximum left atrial volume index; LA ε, left atrial global longitudinal strain. *All *P* values between HCM phenotypes by Bonferroni’s post-hoc test in ANOVA were greater than 0.05 (septal versus mixed, *P* = 0.067; septal versus apical, *P* = 0.150; mixed versus apical, *P* >0.999).

The baseline characteristics of the 79 HCM patients, 79 age- and sex-matched control subjects, and 20 young healthy control subjects are presented in Table 1. The young healthy control group comprised significantly younger subjects and less male subjects compared to the other two groups. However, there were no significant differences between the HCM group and age- and sex-matched control group with respect to age, sex, and body surface area. In the HCM group, 32 subjects (2%) had hypertension and 7 subjects (9%) had diabetes mellitus. Meanwhile, none of the patients in the age- and sex-matched group or young healthy control group had a history of hypertension or diabetes mellitus. When comparing the echocardiographic characteristics, the LA A-P diameter and LA volume indices, including LAVI_max_, LAVI_pre-A_, and LAVI_min_, were significantly increased in the HCM group compared to in the control groups. The trans-mitral flow velocities were comparable, but the annular tissue velocity e’ was significantly lower, and the E/e’ ratio was significantly higher in the HCM group compared to in both control groups. Furthermore, while the reservoir function, as estimated by the LA expansion index, was significantly decreased in the HCM group, the contractile function, as estimated by the LA active emptying fraction, was not significantly different in the HCM group compared to in both control groups. On the other hand, LA ε was significantly decreased in the HCM group compared to in both control groups. When we compared the characteristics by HCM phenotype, there was no significant difference in the LA size and function. Although LA ε was highest in patients with septal HCM, Bonferroni post-hoc analysis did not demonstrate a significant difference between the groups (septal versus apical, *P* = 0.150; septal versus mixed, *P* = 0.067). Fig 2 shows the changes in LAVI_max_ and LA ε by HCM phenotype compared to in the control groups.

**Table 1:** Patient characteristics in the different groups.

				HCM phenotype		
	Young healthy Controls (n = 20)	Age gender matched controls (n = 79)	Entire HCM patients (n = 79)	Apical (n = 32)	Septal (n = 28)	Mixed or diffuse (n = 19)
Age, years	33 ± 5	54 ± 11	54 ± 11*	58 ± 9	53 ± 11	50 ± 14^‡^
Male, n (%)	9 (45)	60 (76)	60 (76) *	24 (75)	22 (79)	14 (74)
Body surface area, m^2^	1.8 ± 2.1	1.7 ± 0.2	1.8 ± 0.2^†^	1.8 ± 0.2	1.7 ± 0.2	1.8 ± 0.2
Heart rate	67 ± 9	66 ± 11	70 ± 11^†^	62 ± 7	60 ± 12	59 ± 15
**Echocardiographic variables**						
LV outflowtrack obstruction, n (%)	−	−	10 (13)	0	8 (29)	2 (11) ^‡^^`^
LV end diastolic diameter, mm	48 ± 4	47 ± 5	44 ± 5*^†^	47 ± 5	41 ± 5	43 ± 6^‡^
LV end systolic diameter, mm	30 ± 4	31 ± 5	27 ± 6^†^	27 ± 3	26 ± 8	28 ± 7
LA A-P diameter, mm	34.2 ± 4.5	36.4 ± 6.0	41.1 ± 6.3*^†^	41.6 ± 5.3	39.1 ± 6.2	43.3 ± 7.4
LAVI_min_, ml/m^2^	13.0 ± 4.0	14.8 ± 5.7	23.4 ± 9.8*^†^	21.5 ± 7.7	22.9 ± 10.3	27.5 ± 11.6
LAVI_pre-A_, ml/m^2^	18.6 ± 4.6	20.9 ± 7.0	32.0 ± 11.7*^†^	29.1 ± 8.3	31.9 ± 13.2	37.1 ± 13.1
LAVI_max_, ml/m^2^	31.3 ± 7.0	30.0 ± 8.7	43.6 ± 15.1*^†^	40.1 ± 11.2	43.1 ± 16.4	50.2 ± 17.2
Transmitral flow E, cm/sec	77.9 ± 16.4	63.6 ± 17.0	62.8 ± 14.7*	63.9± 15.5	63.0 ± 13.3	60.7± 15.9
A, cm/sec	48.0 ± 11.7	63.5 ± 19.4	68.4 ± 22.5*	70.8 ± 22.0	71.0 ± 25.8	60.6 ± 16.7
DT, ms	167 ± 28	201 ± 48	209 ± 59*	194± 46	216± 60	225 ± 72
Tissue Doppler, e’, cm/sec	10.9 ± 1.8	7.6 ± 2.5	5.5 ± 1.9*^†^	5.8 ± 1.8	5.4 ± 2.1	5.1 ± 1.8
a’, cm/sec	7.4 ± 1.5	8.4 ± 1.7	7.7 ± 1.6^†^	7.9 ± 1.6	7.7 ± 1.8	7.4 ± 1.1
s’, cm/sec	7.6 ± 1.3	7.4 ± 1.6	6.9 ± 1.6	7.0 ± 1.5	7.0 ± 1.9	6.8 ± 1.4
E/e’ ratio	7.1 ± 1.1	8.6 ± 2.7	12.8 ± 5.1*^†^	12.0 ± 4.5	13.3 ± 5.7	13.2 ± 5.2
LA expansion index, %	150 ± 57	111 ± 42	94 ± 41*^†^	94 ± 44	94 ± 39	93± 41
LA active emptying fraction, %	30.3 ± 10.5	28.8 ± 11.6	26.9 ± 10.9	26.2 ± 12.1	27.4 ± 11.3	27.3 ± 8.5
LA ε, %	31.5 ± 5.6	27.6 ± 7.7	22.4 ± 7.9*^†^	21.3 ± 6.9	25.3 ± 8.5	20.0 ± 7.5^‡^
**Cardiac MRI variables**						
LV maximum wall thickness, mm	−	−	19.7 ± 4.5	16.7 ± 2.1	21.8 ± 4.8	21.5 ± 4.3^‡^
LV end diastolic volume, ml	160 ± 34	−	140 ± 31*	133 ± 26	142 ± 31	150 ± 39
LV end systolic volume, ml	72 ± 20	−	43 ± 18*	38 ± 13	47 ± 22	44 ± 16
LV ejection fraction, %	55 ± 6	−	70 ± 8*	72 ± 6	68 ± 9	71 ± 7
LV mass index, g/m^2^	43 ± 7	−	75 ± 26*	65 ± 15	75± 25	94 ± 32^‡^
Presence of LGE, n (%)	−	−	62 (78)	20 (63)	24 (86)	18 (95) ^‡^
Extent of LGE, %	−	−	8.2 ± 10.3	5.0 ± 5.7	8.1 ± 10.5	13.6 ± 13.9^‡^

Data are presented as the mean ± SD or n (%). Abbreviations: LV, left ventricle; LA, left atrium; LA A-P diameter, LA maximum anterior-posterior diameter; LAVI_min_, minimum LA volume index; LAVI_pre-A_, LA volume index before atrial contraction; LAVI_max_, maximum LA volume index; DT, deceleration time; LGE, late gadolinium enhancement

*p <0.05 by student’s *t*-test (HCM group vs. young healthy control group)

^†^p <0.05 by paired *t*-test (HCM group vs. age- and sex-matched control group)

^‡^p <0.05 by ANOVA.

To define significant predictors of LA remodeling and dysfunction, we compared the characteristics of HCM patients according to the median values of LAVI_max_ and LA ε in the HCM group (Table 2). When we divided the HCM group by the median LAVI_max,_ 38.7 ml/m^2^, patients with increased LAVI_max_ of > 38.7 ml/m^2^ showed decreased e’ and s’ velocities, and increased E/e’ ratio. Moreover, although the LA expansion index and active emptying fraction were not significantly different, the LA ε was significantly decreased in patients with LAVI_max_ > 38.7 ml/m^2^. The LV maximal wall thickness, evaluated by cardiac MRI, was also not significantly different according to the LAVI_max_ or LA ε. Instead, the LV mass index, evaluated by cardiac MRI, was significantly increased in patients with increased LAVI_max_, whereas the LGE presence and extent were not. When we divided the HCM group by the median LA ε, 21%, patients with decreased LA ε of **≤**21% showed significantly increased LA A-P diameter and volume indices, including LAVI_max_, LAVI_pre-A_, and LAVI_min_. Among the MRI variables, the HCM phenotype and maximal LV wall thickness were not significantly different in patients with decreased LA ε. However, the LV mass index was increased, LGE was observed more frequently, and the LGE extent was significantly increased in patients with decreased LA ε (Table 2).

**Table 2:** Patient characteristics by LAVI_max_ and LA ε.

	LAVI_max_		LA ε	
	LAVI_max_ ≤ 38.7 ml/m^2^ (n = 39)	LAVI_max_ > 38.7 ml/m^2^ (n = 40)	LA ε > 21.0% (n = 39)	LA ε ≤ 21.0% (n = 40)
Age, years	52 ± 11	56 ± 12	53 ± 12	56 ± 11
Men, n (%)	32 (82)	28 (70)	30 (77)	30 (75)
Body surface area, m^2^	1.8 ± 0.2	1.7 ± 0.2	1.8 ± 0.2	1.8 ± 0.2
**Echocardiographic variables**				
LV end diastolic diameter, mm	45 ± 5	43 ± 6	44 ± 5	44 ± 6
LV end systolic diameter, mm	28 ± 5	27 ± 7	25 ± 4	29 ± 7*
LA A-P diameter, mm	38.8 ± 5.4	43.4 ± 6.4*	38.8 ± 5.3	43.4 ± 6.5*
LAVI_min_, ml/m^2^	17.2 ± 4.6	29.6 ± 9.7*	19.4 ± 6.0	27.4 ± 11.2*
LAVI_pre-A_, ml/m^2^	23.5 ± 5.6	40.3 ± 10.0*	26.6 ± 7.9	37.4 ± 12.4*
LAVI_max_, ml/m^2^	31.6 ± 5.4	55.3 ± 12.0*	37.6 ± 11.6	49.5 ± 15.9*
Transmitral flow E, cm/sec	61.7 ± 11.1	63.8 ± 17.7	65.1 ± 13.1	60.6 ± 16.0
A, cm/sec	64.8 ± 20.1	72.0 ± 24.3	69.5 ± 20.8	67.4 ± 24.3
DT, ms	196 ± 47	221 ± 66	199 ± 48	219 ± 67
Tissue Doppler, e’, cm/sec	6.1 ± 1.9	4.8 ± 1.8*	5.9 ± 2.1	5.0 ± 1.6*
a’, cm/sec	8.0 ± 1.3	7.4 ± 1.8	8.2 ± 1.2	7.3 ± 1.8*
s’, cm/sec	7.4 ± 1.5	6.5 ± 1.7*	7.2 ± 1.7	6.6 ± 1.6
E/e’ ratio	10.8 ± 3.1	14.7 ± 6.0*	12.0 ± 3.9	13.5 ± 6.0
LA expansion index, %	91 ± 36	96 ± 46	98 ± 38	90 ± 45
LA active emptying fraction, %	26.6 ± 10.3	27.2 ± 11.6	26.4 ± 10.5	27.4 ± 11.4
LA ε, %	25.0 ± 7.9	19.8± 7.0*	28.5 ± 6.5	16.4 ± 2.7*
**Cardiac MRI variables**				
LV maximum wall thickness, mm	19.0 ± 4.7	20.3 ± 4.2	18.9 ± 5.0	20.4 ± 4.3
LV end diastolic volume, ml	139 ± 33	141 ± 31	138 ± 34	143 ± 29
LV end systolic volume, ml	45 ± 14	41 ± 21	41 ± 15	44 ± 20
LV ejection fraction, %	68 ± 6	72 ± 9	70 ± 7	70 ± 9
LV mass index, g/m2	69 ± 25	82 ± 25*	68 ± 24	82 ± 26*
Presence of LGE, n (%)	28 (72)	34 (85)	27 (69)	35 (88) *
Extent of LGE, %	6.5 ± 9.7	9.8 ± 10.8	4.8 ± 5.4	11.5 ± 12.8*
**HCM phenotype**				
Apical type, n (%)	20 (51)	12 (30)	15 (39)	17 (43)
Septal type, n (%)	13 (33)	15 (38)	18 (46)	10 (25)
Mixed or diffuse type, n (%)	6 (16)	13 (32)	6 (15)	13 (32)

Data are presented as the mean ± SD or n (%)

Abbreviations: LA, left atrium; LAVI_max_, maximum LA volume index; LA ε, LA global longitudinal strain; LV, left ventricle; LA A-P diameter, LA maximum anterior-posterior diameter; LAVI_min_, minimum LA volume index; LAVI_pre-A_, LA volume index before atrial contraction; DT, deceleration time; LGE, late gadolinium enhancement.

^*^P < 0.05 by Student’s *t*-test.

**Fig 3:**
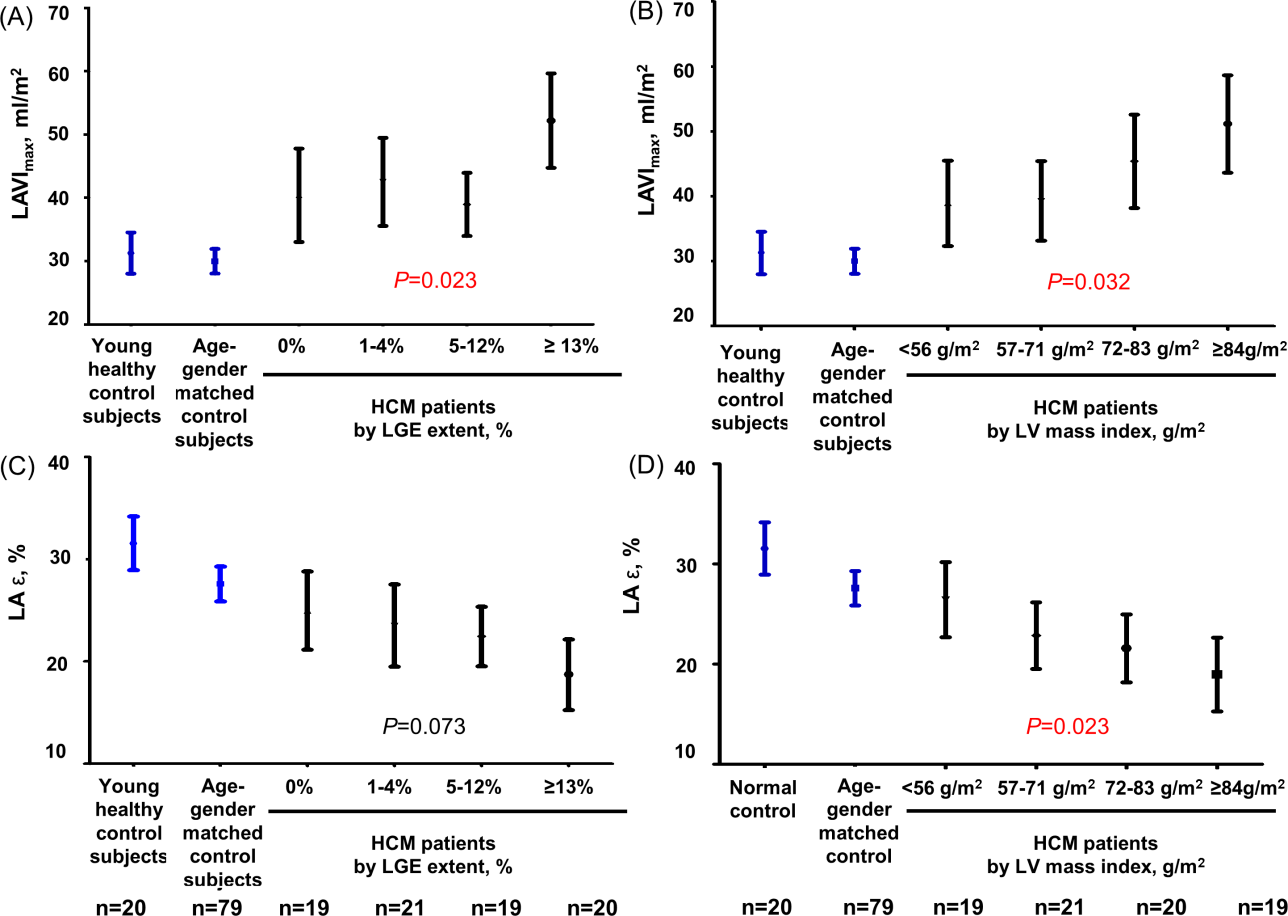
Maximal left atrial volume index (A, B) and global longitudinal strain (C, D) in young healthy control subjects, age- and sex-matched control subjects, and each group of hypertrophic cardiomyopathy stratified by left ventricular late gadolinium enhancement extent and left ventricular myocardial mass index. Abbreviations: LAVI_max_, maximum left atrial volume index; LGE, late gadolinium enhancement; LV, left ventricle; LA ε, left atrial global longitudinal strain.

The associations of LAVI_max_ and LA ε with the LV variables evaluated by cardiac MRI are demonstrated in Table 3. By univariate linear regression analysis, LAVI_max_ was found to be associated with age, presence of LV outflow track obstruction, E/e’ ratio, LA ε, and LV mass index, while LA ε was associated with the LV mass index and LGE extent. However, when we performed multivariate linear regression analyses, only the LV mass index was independently associated with both LAVI_max_ and LA ε. When we illustrated the relation between LAVI_max_ and LA ε according to the LV mass index and the LGE extent by quartiles, both LAVI_max_ and LA ε showed a graded association with the LV mass index, but not with the extent of LGE (Fig 3).

**Table 3:** Univariate and multivariate linear regression analyses of factors associated with LAVI_max_ and LA ε.

	LAVI_max_					LA ε				
	Univariate analysis		Multivariate analysis			Univariate analysis		Multivariate analysis		
	ß	*P* value	ß	*P* value	VIF	ß	*P* value	ß	*P* value	VIF
Age, per 1 years	0.247	0.028	0.203	0.065	1.229	-0.206	0.068	-0.246	0.034	1.229
Male gender	0.173	0.127	0.133	0.220	1.224	-0.146	0.199	-0.206	0.071	1.210
**Echocardiographic variables**										
LV outflow track obstruction	0.289	0.010	0.121	0.266	1.226	0.104	0.366	−	−	−
E/e’	0.422	<0.001	0.216	0.061	1.360	-0.185	0.102	0.019	0.870	1.247
LA expansion index, per 1%	0.042	0.710	−	−	−	0.095	0.406	−	−	−
LA active emptying fraction, per 1%	0.006	0.959	−	−	−	-0.059	0.607	−	−	−
LA ε, per 1%	-0.473	<0.001	−	−	−	-	-	−	−	−
**Cardiac MRI variables**										
Max wall thickness, per 1 mm	0.202	0.074	−	−	−	-0.132	0.247	−	−	−
LV end diastolic volume, per 1 ml	0.054	0.635	−	−	−	-0.038	0.743	−	−	−
LV end systolic volume, per 1 ml	-0.058	0.614	−	−	−	-0.027	0.815	−	−	−
LV ejection fraction, per 1%	0.193	0.088	0.174	0.098	1.140	-0.026	0.839	−	−	−
LV mass index, per 1 g/m^2^	0.273	0.015	0.289	0.014	1.378	-0.288	0.010	-0.341	0.006	1.409
Extent of LGE, per 1%	0.193	0.089	0.107	0.308	1.153	-0.289	0.010	-0.205	0.066	1.149

Abbreviations: LA, left atrium; LAVI_max_, maximum LA volume index; LA ε, LA global longitudinal strain; LV, left ventricle; LGE, late gadolinium enhancement; VIF, variance inflation factor.

## Discussion

The present study demonstrated the following findings: 1) HCM patients showed an increased LA size, impaired reservoir function, and decreased LA ε compared to control subjects. 2) LA remodeling and dysfunction did not significantly differ according to the phenotype of HCM. 3) The LV mass index, evaluated by cardiac MRI, was independently associated with increased LA volume and decreased LA ε, while the LGE extent was not.

Atrial fibrillation is the most common arrhythmia in HCM, occurring in 20% of patients and potentially impacting the prognosis of HCM [2]. Since atrial remodeling is recognized as a key feature in the pathogenesis and perpetuation of atrial fibrillation, evaluating the LA size and mechanical function in HCM patients has prognostic importance [2, 4, 22]. For that reason, there has been an increasing interest in noninvasive evaluations of LA size and mechanical function using 2D echocardiography. However, to date, data on LA enlargement and dysfunction in HCM are sparse [5, 6, 23]. Yang et al. included 104 HCM patients and found that patients with HCM and LA enlargement (LAVI_max_ > 34 ml/m^2^) had more serious cardiovascular events and demonstrated greater LV hypertrophy and more diastolic dysfunction compared to HCM patients without LA enlargement [5]. However, HCM patients with atrial fibrillation were also included in the study, and there was no control group for comparison. Shin et al. performed 3D echocardiography on 26 HCM patients and 15 control subjects and found that the LAVI_max_ was increased and LA active emptying fraction was decreased in HCM patients [6]. However, their study included control subjects without age- and sex-matching, and, in fact, there was a significant age difference between the HCM and control groups, potentially limiting the value of the study findings, as age is an important determinant of LA size and function. To our knowledge, only the study by Tigen et al. evaluated LA volume and function of HCM patients by using 2D echocardiography and compared HCM patients to age- and sex-matched control subjects [23]. The investigators demonstrated that the LAVI was significantly increased and LA reservoir function was significantly decreased in HCM patients compared to control subjects, similar to in the present study. Moreover, they also reported that the LA peak early and late diastolic longitudinal strains measured by 2D speckle-tracking echocardiography were significantly decreased in HCM patients. However, they did not perform cardiac MRI, and LA strain according to the HCM phenotype, LV mass index, LGE presence and extent were not evaluated.

Strain analysis with 2D speckle-tracking echocardiography can be applied to the LA to quantify the magnitude of LA deformation [8, 10, 12]. Although LA functions are traditionally estimated using 2D echocardiography and Doppler analysis of transmitral and pulmonary vein flow, 2D echocardiography is limited by the use of geometric models to determine the volume of a non-symmetric chamber and by errors due to foreshortening. In addition, the evaluation of LA function by Doppler analysis is indirect and therefore also limited. Meanwhile, strain analysis is expected to allow a more direct assessment of LA endocardial contractility and passive deformation. Previous studies have already reported that LA ε is significantly decreased in patients with paroxysmal atrial fibrillation compared to in normal control subjects [10] and that decreased LA ε is associated with atrial fibrillation progression to the persistent or permanent stage [8] and with recurrence after catheter ablation [12]. In our previous study, LA ε was significantly decreased in patients with first paroxysmal atrial fibrillation (27.3 ± 7.2%) compared to age- and sex-matched controls (32.6 ± 7.0%) and was associated with the LA volume index and reservoir function [10]. In a subsequent multicenter study evaluating 313 patients with paroxysmal atrial fibrillation, decreased LA ε was an independent predictor of progression of atrial fibrillation and served as a good predictor even in patients without LA enlargement [8]. In the present study, we first reported a significantly decreased LA ε in HCM patients (22.4 ± 7.9%) compared to age- and sex-matched control subjects (27.6 ± 7.7%) and young healthy control subjects (31.5 ± 5.6%), as well as a significantly increased LA diameter and volume index. These findings support the hypothesis that HCM patients, even with sinus rhythm, might demonstrate LA remodeling and dysfunction before the development of atrial fibrillation, and that LA ε could represent the progression of LA remodeling.

Although HCM patients are usually expected to have increased LA volume and decreased LA mechanical function, it has not been evaluated which characters of HCM determine the progression of LA remodeling and dysfunction. Therefore, in the present study, we also attempted to evaluate the association between HCM characteristics and LA remodeling and dysfunction. First, we defined the subtype of HCM and quantified the LV mass by using cine-MRI. Cine-MRI provides excellent contrast between the blood pool and myocardium, without limitation of either the imaging window or imaging plane, and therefore enables detailed characterization of the HCM phenotype. Further, cine-MRI is highly accurate and allows reproducible quantification of the LV mass [24]. When we divided the HCM patients by the median values of LAVI_max_ and LA ε, the phenotype of HCM was not significantly different in patients with increased LAVI_max_ or in those with decreased LA ε. The LV maximal wall thickness was also not significantly different according to LAVI_max_ or LA ε, whereas the LV mass index was significantly higher in patients with increased LAVI_max_ and in patients with decreased LA ε. In addition, the LV mass index demonstrated graded associations and was independently associated with both LAVI_max_ and LA ε. These observations raise a potentially important consideration for risk stratification of HCM. Although the current risk stratification strategy for HCM uses the maximal LV wall thickness to represent the overall burden of hypertrophy, it has often been proven to be an unreliable estimate of the total LV mass index [24]. The present analysis supports the previous findings that the LV mass index rather than the maximal wall thickness may prove more relevant to the assessment of risk in HCM [24].

Lastly, we also evaluated the presence and extent of LV myocardial fibrosis by using LGE-MRI. LGE evaluated by cardiac MRI is known to correlate with LV wall thickening and to inversely correlate with the LV ejection fraction [25–27]. In addition, it has prognostic value in predicting adverse cardiovascular events, including sudden cardiac death, heart failure death, cardiovascular mortality, and all-cause mortality [15, 28, 29]. Therefore, the assessment of LGE by cardiac MRI is expected to have the potential to provide important information to improve risk stratification in HCM. However, in the present study, the LGE presence and extent were not significantly different in patients according to the LAVI_max_. Although LGE was more frequently observed and the LGE extent was significantly increased in patients with decreased LA ε, the LGE extent failed to demonstrate a graded association with LA ε and was not independently associated with either LAVI_max_ or LA ε when adjusted for the LV mass index. Thus, in contrast to previous observations, these results suggest that the LV mass index rather than the LGE extent is an important determinant of LA remodeling and dysfunction. It is still conceivable that the association between LGE and LAVI_max_ or LA ε might bae alleviated because our study population demonstrated relatively high prevalence of LGE of 78%. Considering that HCM is a heterogeneous cardiac disease with a diverse clinical presentation and course, further studies including larger number of patients are required to confirm our findings and to evaluate whether the LV mass index or LGE extent can predict the occurrence and progression of atrial fibrillation in HCM.

### Study Limitations

First, the study cohort was not population-based, and a relatively small number of patients were enrolled. Thus, the generalizability of our findings might be limited. Although this study is the largest one to evaluate LA ε of HCM patients compared to age- and sex-matched control subjects, we could not match control subjects by hypertension and diabetes which could affect LA size and function. Additionally, the absence of HCM with atrial fibrillation in the HCM group did not allow the evaluation of LA remodeling and dysfunction according to the occurrence of atrial fibrillation. Second, this is a cross-sectional study; thus, we substituted LA enlargement and dysfunction for occurrence of atrial fibrillation. LA enlargement and dysfunction were assessed by LAVI_max_ and LA ε, respectively. Although LAVI_max_ and LA ε are well-known predictors of the occurrence, progression, and recurrence of atrial fibrillation [8, 10, 12], the use of these markers needs to be supported by future studies to confirm that the LA volume and mechanical function, including LA ε, can predict the occurrence of atrial fibrillation in HCM patients. Third, in the present study, we evaluated the LA volume and function only by 2D echocardiography and not by using cine-MRI. As we already discussed, a 2D echocardiographic measure of a non-symmetric chamber is limited in its accuracy. However, the cardiac MRI protocols in our institution do not produce LA short axial images, and thus do not allow 3D volumetric measurements of the LA. Although the biplane area length method is still available, it is also limited by the geometric assumption, similar to 2D echocardiography. Fourth, we also did not measure LA myocardial fibrosis by LGE-MRI. Although LGE-MRI appears to represent a promising tool for assessing LA fibrosis, most previous studies were performed in animal models and came from a few specialized centers. Since the LA wall is significantly thinner compared with the LV wall, obtaining images with sufficient spatial resolution to detect atrial fibrosis in a quantifiable and objective manner remains challenging. Finally, in the present study, we aimed to evaluate LA volume and function, including LA ε, by speckle tracking echocardiography in patients with HCM. Further studies are required to address these issues.

## Conclusions

In this study, we demonstrated that patients with HCM have increased LAVI, impaired reservoir function, and decreased LA ε in comparison with age- and sex-matched control subjects and young healthy control subjects. LA remodeling and dysfunction, as evaluated by LAVI_max,_ and LA ε were associated with the LV mass index. In contrast prior studies, LV myocardial fibrosis by LGE-MRI was not independently associated with LA remodeling and dysfunction.
